# Factors Influencing Retention in Care after Starting Antiretroviral Therapy in a Rural South African Programme

**DOI:** 10.1371/journal.pone.0019201

**Published:** 2011-05-03

**Authors:** Tom H. Boyles, Lynne S. Wilkinson, Rory Leisegang, Gary Maartens

**Affiliations:** 1 Madwaleni Hospital, Eastern Cape, South Africa; 2 Division of Infectious Diseases and HIV Medicine, Department of Medicine, University of Cape Town, Cape Town, South Africa; 3 Division of Clinical Pharmacology, Department of Medicine, University of Cape Town, Cape Town, South Africa; Institute of Infectious Diseases and Molecular Medicine, South Africa

## Abstract

**Introduction:**

The prognosis of patients with HIV in Africa has improved with the widespread use of antiretroviral therapy (ART) but these successes are threatened by low rates of long-term retention in care. There are limited data on predictors of retention in care, particularly from rural sites.

**Methods:**

Prospective cohort analysis of outcome measures in adults from a rural HIV care programme in Madwaleni, Eastern Cape, South Africa. The ART programme operates from Madwaleni hospital and seven primary care feeder clinics with full integration between inpatient and outpatient services. Outreach workers conducted home visits for defaulters.

**Results:**

1803 adults initiated ART from June 2005 to May 2009. At the end of the study period 82.4% were in active care or had transferred elsewhere, 11.1% had died and 6.5% were lost to follow-up (LTFU). Independent predictors associated with an increased risk of LTFU were CD4 nadir >200, initiating ART as an inpatient or while pregnant, and younger age, while being in care for >6 months before initiating ART was associated with a reduced risk. Independent factors associated with an increased risk of mortality were baseline CD4 count <50 and initiating ART as an inpatient, while being in care for >6 months before initiating ART and initiating ART while pregnant were associated with a reduced risk.

**Conclusions:**

Serving a socioeconomically deprived rural population is not a barrier to successful ART delivery. Patients initiating ART while pregnant and inpatients may require additional counselling and support to reduce LTFU. Providing HIV care for patients not yet eligible for ART may be protective against being LTFU and dying after ART initiation.

## Introduction

Data from multiple sources show that antiretroviral treatment (ART) dramatically improves the prognosis of individuals with HIV infection and universal access to ART is now accepted as an important part of the integrated response to the epidemic in all settings [1]–[7]. It has become increasingly clear that as access to ART improves the success of services at a population level will primarily be determined not by issues of drug efficacy but rather by the effectiveness of drug delivery programmes [8], [9].

A major barrier to the success of ART programmes in Africa may be low rates of long-term retention in care. A systematic review of ART programmes in sub-Saharan Africa found that on average only 64% of patients who initiated ART remain in care after 3 years [10]. A recent analysis of 44 177 patients attending public sector ART services in South Africa found that 71% of patients remained in care after 24 months and 59.6% after 48 months with the proportion of these being loss to follow-up (LTFU) increasing over that time [11]. LTFU is the commonest cause of attrition, followed by death, which is often under-estimated [10].

Despite the increasing allocation of resources to expand access to ART in sub-Saharan Africa, little is known about how best to deliver treatment services and in particular how to improve rates of long-term retention in care. There is a particular lack of outcome data from ART programmes in low resource rural areas. The suggestion has also been made that programmes with high retention rates should serve as models for future improvements [12].

The aims of our study were to determine the factors predicting loss to follow-up and mortality in a public-sector HIV and ART programme in rural South Africa.

## Methods

### Setting

The Madwaleni HIV wellness and ART programme operates at Madwaleni Hospital and its 7 primary healthcare feeder clinics. It is situated in the deeply rural Elliotdale/Xora area of the Mbhashe sub-district in the Eastern Cape, which is one of the most socio-economically deprived magisterial districts in South Africa [13]. The population is almost exclusively black African and Xhosa speaking with 90% of people living below the poverty line [13]. Catchment population is estimated to be 80 000–100 000 of whom 60% are adult [14]. HIV prevalence in the Eastern Cape was estimated in 2005 to be 15.2% in 15–49 years olds [15]. The programme is predominantly funded by the South African government with donor support from Aurum Institute for Health Research and Donald Woods' Foundation. Stipends were paid to peer educators and no co-payment was required from patients for any component of care.

### Programme enrolment

Patients testing positive for HIV at one of the healthcare facilities or at the hospital's HIV counselling and testing community based outreach programme were counselled to join the weekly HIV support groups led by peer educators at one of the 7 feeder clinics. Outpatients were enrolled at their third visit and pregnant women at their first visit. There was an integrated inpatient service conducted at the bed-side and inpatients were enrolled after their second weekly counselling session. Inpatients not commencing ART while in hospital were referred to the outpatient programme upon discharge.

### Pre-ART care

Patients who were not eligible for ART were encouraged to attend support groups at feeder clinics 2 weekly for the first 6 months and monthly thereafter. At each visit they received symptom screening by trained lay counsellors for tuberculosis and sexually transmitted infections and were referred to a nurse if symptomatic. Prophylactic co-trimoxazole was prescribed if patients had a CD4 count <500 cells/µl or a WHO stage 2, 3 or 4 illness. Women were offered cervical cancer screening. Isoniazid preventive treatment was introduced in December 2008 for patients without symptoms of active tuberculosis (tuberculin skin tests were not performed). All patients received multivitamins and nutritional supplements were provided if their body mass index was <18.5 kg/m^2^. CD4 count was measured 6 monthly until patients were eligible for ART.

### ART initiation

Eligibility criteria for public sector patients in South Africa were the same as the 2003 WHO guidelines for resource limited settings (CD4 lymphocyte count <200 cells/µl or WHO stage 4 disease) [16], [17]. Eligible patients were enrolled in an ART preparation programme. Pill counts of co-trimoxazole and/or multivitamins were done at weekly intervals to assess treatment readiness, inpatients self administered prescribed medication for this purpose. Patients who were ineligible for ART underwent this process as part of pre-ART care. Individual ART adherence counselling by trained peer educators was done after 3 accurate pill counts for outpatients or 2 accurate pill counts for inpatients but accelerated at the discretion of the doctor in clinically unstable patients. Treatment supporters were sought for patients who were unable to accurately complete pill counts. Outpatients received a preparatory home visit and disclosure to household members was strongly encouraged. All patients who were eligible for ART but had not initiated therapy were discussed at a weekly team meeting and followed-up by peer educators by phone call or home visit.

Once prepared, all patients were scheduled to start ART within 7 days and at no point was there a waiting list to begin treatment. On the day of ART initiation all patients were clinically evaluated by a doctor who made the final decision to prescribe ART. A second session of individual ART adherence was conducted by a pharmacy assistant. ART initiation was temporarily deferred when patient understanding was inadequate in which case adherence counselling was repeated and extra social support added whenever possible. ART was also deferred in order to investigate or treat opportunistic infections as outlined in national treatment guidelines. Inpatient ART was self administered except in those whose clinical condition precluded adequate counselling (accelerated inpatient group). The latter group of inpatients were prepared for self administered ART before hospital discharge.

### Follow-up

All patients were followed up 2 weeks after ART initiation to monitor tolerability and adherence and were then referred back to the HIV clinic at the feeder clinics for monthly visits for the first year on ART. After one year adherent patients with an undetectable viral load were given an option of 3 monthly visits.

All patients who were more than 7 days late for collecting a prescription were contacted telephonically (seldom available), otherwise visited at home by an outreach worker.

### Task shifting and decentralisation

With increasing patient load exceeding the expansion in clinical staff the programme focused on extensive task shifting. As a result, while patients initiating ART early in the programme saw a doctor at each follow-up visit for the first year, those initiating ART in later years received much of their follow-up clinical care from nurses. All patients had access to doctor level care either by referral from clinic nurses or by self-referral to the hospital outpatients department.

The programme model sought to decentralise care to the primary healthcare clinics as much as possible. As a result patients received every aspect of their care at their closest clinic with the exception of the day of ART initiation and the 2 week follow-up visit which took place at the hospital.

### Laboratory testing

Baseline blood tests when joining the programme were HIV ELISA, CD4 count, full blood count, syphilis serology and alanine aminotransferase (ALT). These were repeated at ART initiation (except HIV ELISA) if not taken within the preceding 3 months. Viral load was measured at ART initiation until May 2009 when it was removed from the national programme. ALT was monitored after 2, 4, 8 & 12 weeks for patients initiating nevirapine. CD4 count, viral load and full blood count were taken every 24 weeks after ART initiation.

### Regimens

First-line ART comprised stavudine (D4T), lamivudine (3TC) plus a non-nucleoside reverse transcriptase inhibitor (efavirenz or nevirapine). The dose of D4T was 30 mg for those <60 kg and 40 mg for those >60 kg until January 2007 when all patients received D4T 30 mg regardless of weight. The second-line regimen for those failing the first-line treatment comprised lopinavir/ritonavir, zidovudine and didanosine. Decisions to change treatment following step up adherence counselling were made by a doctor. Co-trimoxazole prophylaxis was stopped when the CD4 count was >350 cells/µl. The programme experienced no interruptions in drug supply during the study period.

### Ethics

Ethical approval for this study was obtained from the University of Cape Town Research Ethics Committee. The need for informed consent from patients was waived as the study represented enhanced surveillance of data collected as part of routine patient care.

### Data analysis

Patient enrolment began in January 2005 and the first patients started ART in June 2005. We included all patients who initiated ART before the end of May 2009; the end of the study period was 31 August 2009 to allow for at least 3 months of follow-up data on ART. All data were prospectively entered into a locally developed electronic database which used Microsoft Access as the frontend and Microsoft Sequel Server 2005 Express as the backend (Microsoft Corporation, Virginia, USA). Paper records containing the data along with supplementary clinical information were kept separately. For the analysis, data storage, basic calculations and data extraction was handled in Microsoft Sequel Server 2008. Outcomes analysis was performed in Stata 11.

Frequency distribution and median with interquartile range (IQR) were used to describe the variables because the distribution of the continuous variables was found to be non-normal. The month in which patients started ART was set as month zero. One day of follow-up was added to patients who did not attend follow-up after ART initiation for survival analyses.

Multiple Cox proportional hazard regression analysis, adjusted for competing risks, was used to identify variables associated with a likelihood of being LTFU or dying. LTFU was defined as no patient contact for more than 6 months before the end of study period. Virological suppression was defined as <400 copies/ml. Patients transferring to other centres or LTFU were censored at the date of their last contact. Variable estimates and their significance at the 95% level were assessed with robust standard errors.

## Results

Between January 2005 and May 2009, 3411 adults (>19 years) were enrolled into the programme. Ninety seven of the 1900 initiating ART by the end of the study period were ART experienced before enrolling into the programme and were excluded from further analyses. Baseline characteristics and status at the end of the study period of the 1803 ART naïve adults are summarised in table 1. Median follow-up time on ART was 13.3 months (IQR 5.4 to 25.0) and median time from registration to starting ART was 2 months (IQR 1.0 to 4.5). End of study outcomes for the combined group were 71.5% current, 11.1% deceased, 6.5% LTFU and 10.9% transferred. For women initiating ART when pregnant outcomes were 74.5%, 1.1%, 13.6% and 10.9% respectively. For those initiating ART as inpatients 44.5%, 32.4%, 9.7% and 13.4% respectively, for those taking TB treatment when initiating ART 61.4%, 16.3%, 6.9% and 10.8% respectively, for those receiving pre-ART care for >6 months 85.6%, 4.4%, 3.7% and 6.3% respectively and for the 68 patients in the accelerated inpatient group 28%, 52%, 11% and 11% respectively.

**Table 1:** Baseline and end of study characteristics of all patients, and specific patient groups.

	All	Pregnant when initiating ART	Inpatient when initiating ART	Taking TB treatment when initiating ART	>6 months pre-ART care
Number (%)	**1803**	**184 (10.2)**	**247 (13.7)**	**583 (32.3)**	**270 (15.0)**
Age (years) baseline					
Median	**31.9**	**27.1**	**32.8**	**32.3**	**31.0**
IQR	**(26.8–39.1)**	**(23.4–31.5)**	**(28.2–39.6)**	**(27.7–39.7)**	**(26.3–39.0)**
Unknown	**179**	**4**	**45**	**73**	**31**
Sex (%)					
Female	**68.3**	**100.0**	**61.3**	**60.1**	**81.1**
Unknown	**9**	**0**	**7**	**0**	**0**
CD4 count (cells/µl) baseline					
Median	**123**	**191**	**50**	**76**	**192**
IQR	**(55–184)**	**(122–248)**	**(21–116)**	**(35–150)**	**(156–237)**
Unknown	**106**	**18**	**42**	**38**	**9**
Viral load (log_10_) baseline					
Median	**4.8**	**4.3**	**5.3**	**5.1**	**4.5**
IQR	**(4.3–5.4)**	**(3.5–4.7)**	**(4.7–5.8)**	**(4.5–5.6**	**(3.9–5.0)**
unknown	**436**	**52**	**107**	**39**	**56**
Status at end of study (%)					
Current	**71.5**	**74.5**	**44.5**	**61.4**	**85.6**
Deceased	**11.1**	**1.1**	**32.4**	**16.3**	**4.4**
LTFU	**6.5**	**13.6**	**9.7**	**6.9**	**3.7**
Transferred	**10.9**	**10.9**	**13.4**	**10.8**	**6.3**

**Figure 1:**
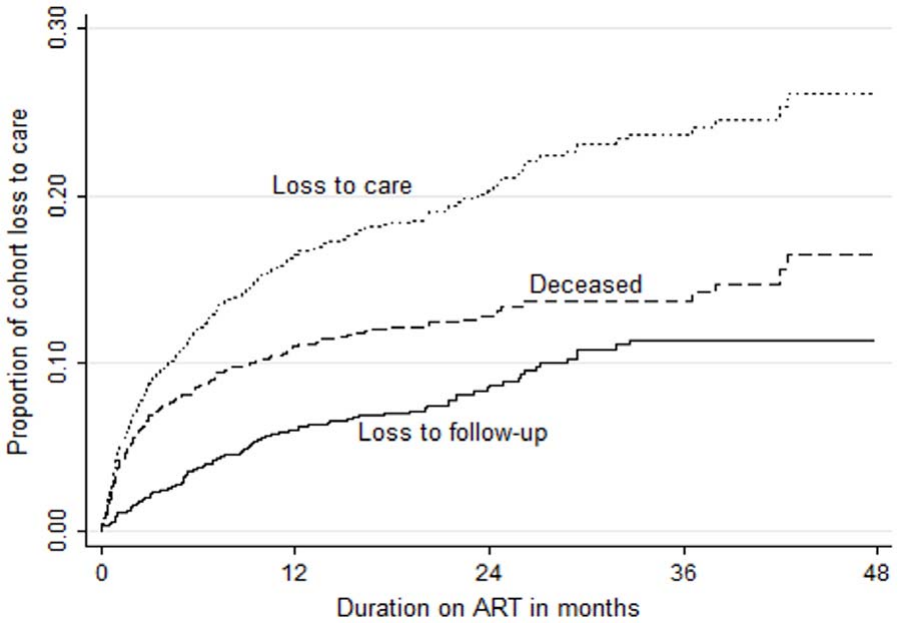
Kaplan-Meier estimates of LTFU, mortality and loss to care by months of treatment with ART for 1803 ART naive adults.

Two hundred and seventy patients who initiated ART had at least 6 months of pre-ART care; their median CD4 count was 304 (IQR 252 to 386) at enrolment and 192 (IQR 156 to 237) at ART initiation. The non-nucleoside reverse transcriptase inhibitor used in first line regimens was efavirenz in 85% and nevirapine in 15%. Thirteen patients switched to second line ART after a median of 16.8 months (IQR 15.6 to 24.1) on first line therapy. Kaplan-Meier estimates of LTFU, mortality and retention in care for all patients are shown in figure 1. After 48 months Kaplan-Meier estimated LTFU was 11.4%, mortality 16.5%, and loss to care 26.1%. There was a relatively constant attrition rate from LTFU over the first 36 months whereas mortality was greater in the first year than subsequent years.

**Figure 2:**
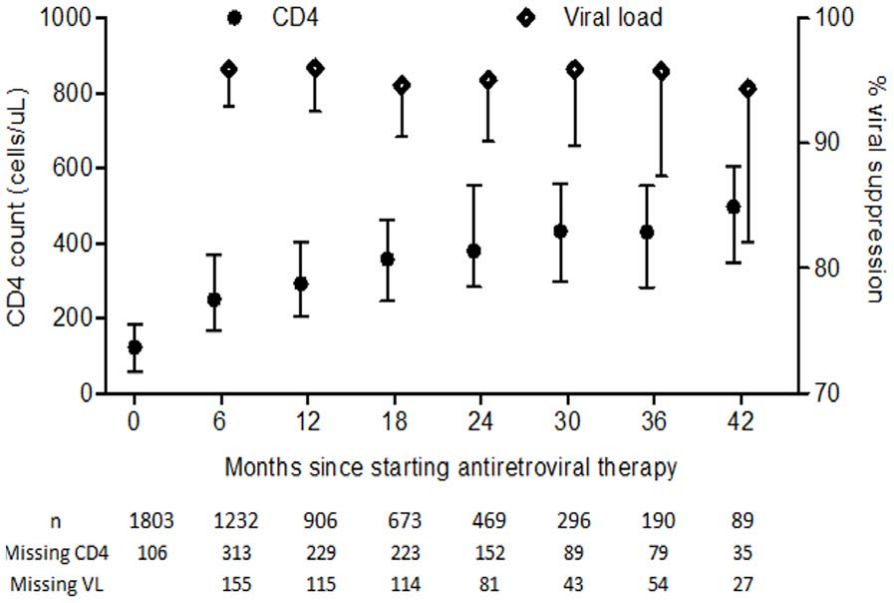
Median CD4 cell count (bars indicate interquartile range) and proportion with HIV viral load suppressed (bar indicates lower 95% confidence interval) at 6 monthly intervals.

CD4 count and viral suppression rates at 6 monthly intervals are shown in figure 2. CD4 counts were more likely to be missing than viral loads due to intermittent difficulties in transporting specimens to the laboratory (CD4 counts need to be processed more rapidly than viral loads after venepuncture). Cox proportional hazard ratios for death and LTFU according to baseline parameters are shown in tables 2 and 3 respectively. Risk of dying was independently associated with lower CD4 counts and starting ART as an inpatient. Being pregnant when starting ART and receiving >6 months pre-ART care were associated with decreased risk of dying. Risk of becoming lost to follow-up was independently associated with higher CD4 count, younger age, starting ART as an inpatient and starting ART while pregnant. Receiving >6 months pre-ART care was associated with a decreased risk of becoming lost to follow-up. There was a time dependent association between risk of death and being an inpatient when starting ART, with increased mortality in the first month (data not shown). We therefore conducted a sub-analysis excluding deaths in the first month. The association between death and starting ART as an inpatient was weakened but remained significant (adjusted hazard ratio 2.15, 95% confidence interval 1.28 to 3.62).

**Table 2:** Cox proportional hazard ratio of risk of dying.

Variables		Hazard Ratio (95% confidence intervals)	p-value
Baseline CD4 count (cells/µl)	0–4950–199≥200	1.76 (1.23 to 2.52)Referent1.17 (0.73 to 1.86)	0.002NA0.515
Sex	MaleFemale	Referent1.20 (0.87 to 1.66)	NA0.279
Age (years)	<2525–50>50	0.69 (0.39 to 1.24)Referent1.33 (0.92 to 1.93)	0.214NA0.132
Starting ART as an inpatient	YesNo	2.91 (1.90 to 4.47)Referent	<0.001NA
Starting ART on tuberculosis treatment	YesNo	1.15 (0.77 to 1.72)Referent	0.494NA
Starting ART in pregnancy	YesNo	0.18 (0.04 to 0.74)Referent	<0.001NA
Pre-ART care >6 months	YesNo	0.50 (0.26 to 0.96)Referent	0.037**NA**

**Table 3:** Cox proportional hazard ratio of risk of loss to follow-up.

Variables		Hazard Ratio (95% confidence intervals)	p-value
Baseline CD4 count (cells/µl)	0–4950–199≥200	1.00 (0.61 to 1.64)Referent1.74 (1.09 to 2.78)	0.991NA0.019
Sex	MaleFemale	Referent1.42 (0.90 to 2.23)	NA0.134
Age (years)	<2525–50>50	1.87 (1.15 to 3.05)Referent1.2 (0.70 to 2.06)	0.012**NA**0.51
Starting ART as an inpatient	YesNo	1.89 (1.06 to 3.38)Referent	0.032**NA**
Starting ART on tuberculosis treatment	YesNo	1.03 (0.63 to 1.68)Referent	0.904**NA**
Starting ART in pregnancy	YesNo	3.20 (1.86 to 5.51)Referent	<0.001**NA**
Pre-ART care >6 months	YesNo	0.49 (0.24 to 1.00)Referent	0.05**NA**

## Discussion

Our study shows that serving a highly socio-economically deprived rural population is not a barrier to successful ART delivery. Estimated four year loss to follow-up rates reported from resource limited settings have ranged from around 19 to 33% [10], [11]. To our knowledge the estimated loss to follow-up rate of 11.4% at four years is lower than any reported from a resource limited setting. A major strength of our programme is that it fully integrates care for inpatients, pregnant women, and patients not yet eligible for ART. Despite higher overall mortality, particularly in the first month, we show that patients can be successfully started on ART while in hospital, but rates of LTFU are higher. Women initiating ART while pregnant have a significant survival advantage although they are at greater risk of becoming LTFU. Importantly, patients with >6 months pre-ART care have both decreased mortality and LTFU after starting ART.

The low rate of LTFU in the Madwaleni programme may be explained by the inclusion of many factors known to reduce LTFU. Proven effective strategies include improved patient preparation [18], use of treatment supporters [19], reduced patient costs [20], improved databases [21] and community support [22]. Our model includes at least 3 individual counselling sessions before starting ART with ongoing group education at support groups. Almost all patients had treatments supporters, decentralisation of services to peripheral clinics reduced patient costs, and an electronic database allowed prompt identification of treatment defaulters who were actively followed-up by phone and home visit.

The formation of active support groups within the community was likely an important factor in the overall success of the model. Importantly, support groups included patients not yet eligible for ART allowing group numbers to increase more quickly and possibly reduce HIV related stigma in the community. The community network of HIV positive people was then able to identify at risk patients and inform staff members of problems.

Our estimated 4 year mortality was higher than in other South African ART programmes [11]. A likely explanation for the higher mortality in our cohort is the inclusion of 247 inpatients starting ART who accounted for approximately 40% of all deaths. Patients who require hospitalisation typically have advanced disease and many would die before starting ART in other programmes and therefore be excluded from mortality estimates. Another possibility is that the community network allowed better ascertainment of death than in other programmes where many patients who are listed as LTFU may have died.

A number of factors could account for the higher rate of LTFU in inpatients and pregnant women in our cohort. Firstly, accelerated ART initiation reduces both the time patients have to prepare for treatment and the number of counselling and support group sessions they can attend. Secondly, patients who test for HIV because of illness, as occurred in most of our inpatients starting ART, or those attending for non-HIV related reasons such as pregnancy are more likely to be non-adherent than those testing because of possible exposure to HIV [23]. Thirdly, depression, which is known to impair adherence to ART [24], is more common in patients with symptomatic than asymptomatic HIV [25]. The prevalence of depression is very high during pregnancy and in the postnatal period in sub-Saharan Africa, particularly in women who know they are HIV-infected [26]–[28]. Women may have less reason to attend a health facility post delivery and need extra time at home to care for the newborn while the inpatients may have been drawn from a wider geographical area due to the severity of their illness and have had further to travel for care after discharge. The increased rate of LTFU in patients with CD4 nadir >200 we observed in our cohort was also reported from another South African ART programme [29]. Under South African guidelines pertaining at the time of our study patients with CD4 >200 were eligible for ART only if they had a WHO stage 4 illness. Therefore, patients in our cohort with CD4 nadir >200 were symptomatic and their higher rate of LTFU might be due the same explanations proposed above for patients starting ART as inpatients.

Although some data are conflicting [30] most studies of women starting ART when pregnant have found an increase in LTFU [31], [32]. The same studies mostly failed to show a difference in overall survival [30], [32] although a small survival advantage was found in a single study [31]. Our data suggest that being pregnant when initiating ART is associated with increased LTFU and decreased mortality. One explanation for the lower mortality we observed in women starting ART when pregnant is that CD4 counts are transiently lowered in pregnancy due to haemodilution [33]. Therefore controlling for CD4 counts at ART initiation in our Cox proportional hazards model of survival confers a survival benefit to pregnant women.

Our finding that patients with >6 months pre-ART care had decreased mortality and LTFU after starting ART suggests that providing comprehensive follow-up care for patients not yet eligible for ART will improve outcomes. Many African ART programmes focus most or all of their time on patients who need ART. Data from the private sector in South Africa showed that patients with >6 months CD4 monitoring prior to ART initiation incurred lower direct costs and had decreased mortality [34], [35].

A limitation of our study, common to all observational studies, is that despite adjustment for potential confounders the possibility of residual confounding exists. The definition we used of LTFU was the same as that used in comparable studies [11], [36]. However, this definition takes no account of unstructured treatment interruptions before the last 6 months of the study period. Civil identification numbers were not widely available and therefore cross referencing with the national death registry was not possible. Ascertainment of death when not observed in our hospital was often based on information from the community. However, we believe this information was fairly complete as it was based on information obtained from home visits by outreach workers. Our model used additional donor funding which may limit the ability to generalize our findings to other settings. However, the programme is primarily state funded to ensure sustainability and while donor funding was important in establishing the model it was only accepted for costs that could feasibly be replaced by state funding in future.

More data are required to better understand the reasons for higher rates of LTFU in patients starting ART during pregnancy or as inpatients, in order to develop strategies to reduce LTFU. Our data suggest that pre-ART care improves outcomes but further analysis of both the costs and benefits of providing comprehensive HIV care services is required.

In summary, the success of ART delivery in Africa is threatened by low rates of retention in care and particularly by high rates of LTFU. Our study shows that low rates of LTFU are achievable, even in remote rural areas, by focusing on activities proven to decrease loss to follow-up, by building a strong community support network, and by introducing active pre-ART care. ART programmes in Africa may be able to improve rates of retention in care by adapting this programme model to their own settings.
